# The roles of long non-coding RNAs in lung cancer

**DOI:** 10.7150/jca.65031

**Published:** 2022-01-01

**Authors:** Zhe Cao, Linda Oyang, Xia Luo, Longzheng Xia, Jiaqi Hu, Jinguan Lin, Shiming Tan, Yanyan Tang, Yujuan Zhou, Deliang Cao, Qianjin Liao

**Affiliations:** 1 Hunan Key Laboratory of Cancer Metabolism, Hunan Cancer Hospital and the Affiliated Cancer Hospital of Xiangya School of Medicine, Central South University, Changsha, 410013, Hunan, China.; 2 Clinical Research Center for Wound Healing in Hunan Province, Changsha 410013, Hunan, China.

**Keywords:** LncRNAs, lung cancer, tumor biomarkers, MALAT1, and HOTAIR

## Abstract

Lung cancer is the most common malignancy, being a serious threat of human lives. The incidence and mortality of lung cancer has been increasing rapidly in the past decades. Although the development of new therapeutic modes, such as target therapy, the overall survival rate of lung cancer remains low. It is urgent to advance the understanding of molecular oncology and find novel biomarkers and targets for the early diagnosis, treatment, and prognostic prediction of lung cancer. Long non-coding RNAs (lncRNAs) are non-protein coding RNA transcripts that are more than 200 nucleotides in length. LncRNAs exert diverse biological functions by regulating gene expressions at transcriptional, translational, and post-translational levels. In the past decade, it has been shown that lncRNAs are extensively involved in the pathogenesis of various diseases, including lung cancer. In this review, we highlighted the lncRNAs characterized in lung cancer and discussed their translational potential in lung cancer clinics.

## 1. Introduction

Lung cancer is the most common malignancy with the highest incidence and mortality among human cancers. Pathologically, lung cancer is sorted into small cell lung cancer (SCLC) and non-small cell lung cancer (NSCLC), and the NSCLC accounts for approximately 85% 1. According to the pathological characteristics, NSCLC is subdivided into three types: lung adenocarcinoma (LAD), large cell carcinoma (LCC) and lung squamous cell carcinoma (LSCC) 2, 3. In recent years, targeted therapy against lung cancer is widely used in clinics, such as tyrosine kinase inhibitors (TKIs) and epidermal growth factor receptor (EGFR) inhibitors 4, 5, but the five-year survival rate of lung cancer is still low due to the late-stage diagnosis and metastasis, as well as drug resistance 6.

Cancer is a complex disease derived from accumulations of genetic and epigenetic alterations, including gene amplification, mutation and abnormal expression, as well as histone methylation and chromosomal modifications 7-9. A wealth of compelling evidence has suggested that aberrantly expressed non-long coding RNAs (lncRNAs) may be important molecules in the pathogenesis and progression of cancers, including lung cancer, and be potential biomarkers for diagnosis, treatment and prognosis of cancers, as well as individualized therapies 10, 11.

The involvement of lncRNAs in the development and progression of lung cancer has attracted the eyeballs of scientists in lung cancer research, and many lncRNAs have been identified and extensively investigated in lung cancer thus far 12-14. Reviews published in lncRNAs in lung cancer include articles focused on single lncRNA in multiple tumors 15-18, as well as briefly on the lncRNA profiles in lung cancer 19, 20. LncRNAs that may act in signaling transduction, diagnosis, therapeutics and prognosis are also comprehensively discussed in review articles 13, 21, 22. Table 1 summarizes the lncRNAs that function in etiology, diagnosis, treatment and prognosis of lung cancer. This present article is aimed to review the lncRNAs in lung cancer from a different angle, highlighting important oncogenic and tumor suppressive lncRNAs characterized in lung cancer. The lncRNAs discussed in this article include lncRNAs MALAT1, HOTAIR, H19, ANRIL, AFAP1-AS1, UCA1, MEG3, GAS5, TUG1, etc. (Table 2).

## 2. LncRNA biology and function

Long non-coding RNAs (lncRNAs) are non-protein coding RNA transcripts that are more than 200 nucleotides in length. Only 1.5% of nucleic acids in a human genome are used for protein encoding; other 98.5% of the genome does not encode proteins 23. LncRNAs as non-protein-coding RNAs were initially considered to be the by-products of the transcription process 24. With the in-depth of knowledge of lncRNAs, the mystery is gradually unveiled. LncRNAs are biologically functional molecules; and according to the relative location to protein encoding genes in the genome, lncRNAs are classified as sense lncRNAs, antisense lncRNAs, bidirectional lncRNAs, intron lncRNAs, intergenic lncRNAs, and enhancer intergenic lncRNAs 25-27.

To date, lncRNAs have been recognized as key regulatory factors in cancer development and progression. They can regulate gene expression at the epigenetic, transcriptional, translational and post-transcriptional levels 27, 28. The most important feature of lncRNA-mediated regulatory network is that lncRNAs can act as a scaffold through which lncRNAs interact with various signaling molecules and regulatory factors. Depending on the type and number of their bond partners, lncRNAs perform a variety of regulatory functions, including gene expression, histone methylation, genomic imprinting, and chromatin modifications 29, 30. LncRNAs may also regulate gene expression through the function as a co-factor of transcriptional factor and a regulator of RNA polymerase II activity or the transcription machinery 31, 32. Via specific complementary interactions with target sequences, lncRNAs can also regulate the post-transcriptional processing and translation of mRNAs, such as capping, splicing, editing, transportation and stability 27, 31. Therefore, lncRNAs are involved in various physiological and pathological processes of the body, including tumorous and non-neoplastic diseases 26, 27, 33.

Aberrant expression of lncRNAs is associated with various cancers. Through regulation of gene expression and signaling transduction, lncRNAs function as oncogenes or tumor suppressors, extensively involved in the development and progression of cancers. They can affect various aspects of cell activities, such as growth and proliferation, survival, migration and invasion, as well as genomic stability. Thus it is increasingly evidenced that lncRNAs play a key role in tumor growth, lymph node/distant metastasis, and patient survival 22, and the single nucleotide polymorphisms (SNP) of lncRNAs are identified as risk factors and correlated to tumorigenesis and metastasis of cancers 34, 35. More interestingly, some lncRNAs are found to be increased in the plasma of cancer patients, yielding the possibility as diagnostic markers in blood 36, 37. Therefore, the identification and characterization of lncRNAs have opened a new window in the management of cancers. They may serve as biomarkers of cancers for the development of novel diagnostic tools and prediction of prognosis and as potential targets for novel strategies of cancer therapy. LncRNAs are also involved in therapeutic resistance (e.g. chemo- or radiological resistance) of cancers and may thus be is potential targets to improve the treatment efficacy of cancers 38, 39. In summary, lncRNAs are important players in the development and progression of cancers, including lung cancer; the discovery and characterization of novel lncRNAs provide new platforms for the development of novel management strategies of cancers.

## 3. Oncogenic lncRNAs in lung cancer

### 3.1. Metastasis-Associated Lung Adenocarcinoma Transcript (MALAT1)

MALAT1 is an 8.7 kb intergenic lncRNA located on chromosome 11q13.1. MALAT1 is expressed in a variety of human tissues and is evolutionarily conserved in mammals 40. MALAT1 is involved in post-transcriptional regulation of gene expression and mRNA splicing, and is associated with the development of a variety of tumors, including lung cancer, liver cancer, prostate cancer, colon cancer, uterus cancer, ovarian cancer, breast cancer, neuroblastoma, and hematological malignancies 41-45.

MALAT1 is an oncogenic lncRNA promoting cancer cell proliferation, migration, invasion, epithelial-mesenchymal transition (EMT) and chemoresistance 43, 45, 46. In NSCLC tissues, the expression of MALAT1 is higher than that in adjacent normal tissues, and the MALAT1 expression is correlated with the overall survival of NSCLC 47, and in lung cancer patients, MALAT1 may negatively regulate the myeloid-derived suppressor cells (MDSCs) 48. In cultured NSCLC cells, silencing of MALAT1 inhibits cell proliferation and colony formation 49.

**Figure 1:**
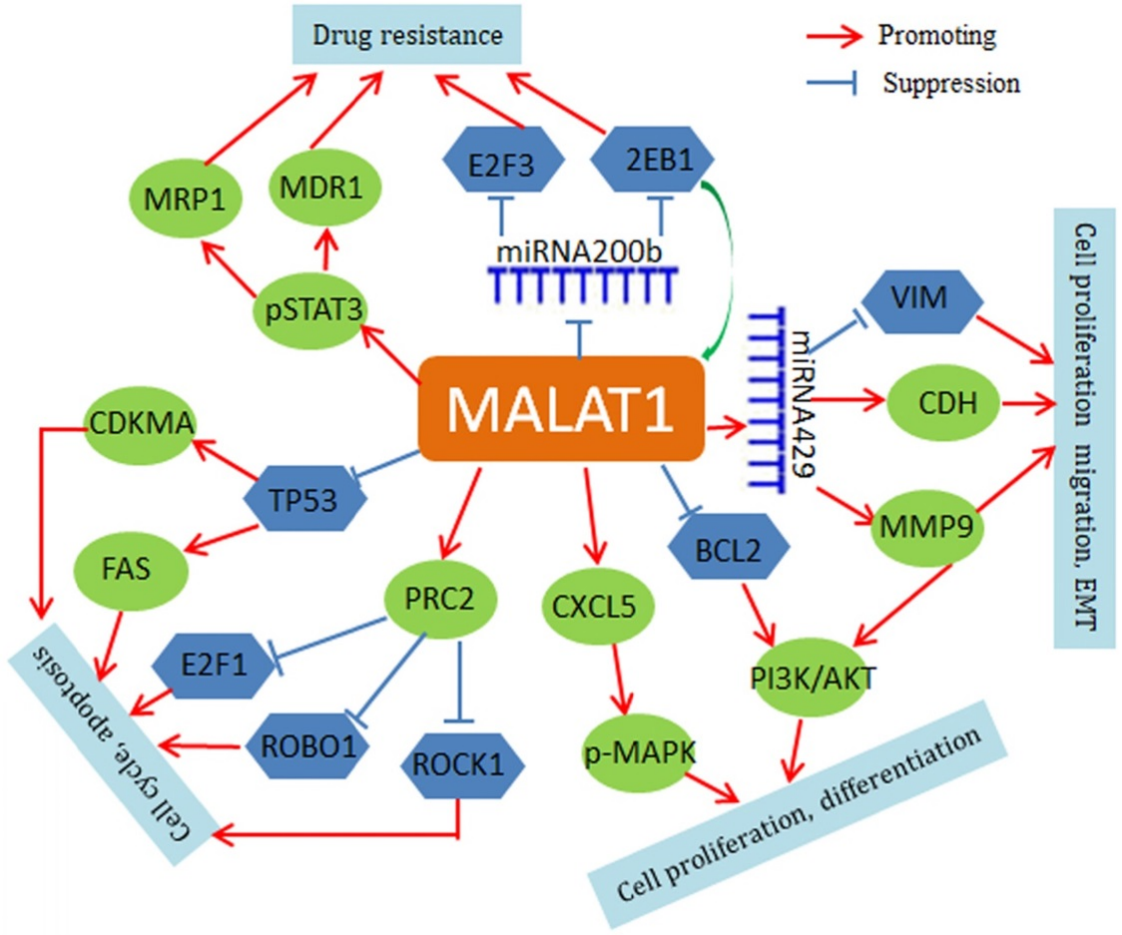
** Regulatory network of MALAT1 in lung cancer.** MALAT1 regulates FAS and CDKMA through inhibition of TP53. Through a miRNA429-mediated mechanism, MALAT1 regulates VIM and MMP9. MALAT1 activates PRC2 expression to inhibit E2F1, ROBO1 and ROCK1. MALAT1 also up-regulates phospho-STAT3, ABCB1 and MRP1. In addition, MAIAT1 can acts as a ceRNA for regulation of miRNAs, such as miR-429 and miR-200b.

Mechanistically, MALAT1 negatively regulates p53 promoter activity in NSCLC cell lines (A549 and H1299), and MALAT1 depletion leads to up-regulation of both p21 and FAS and cell cycle arrest in G1 50, 51. MALAT1 also modulates VIM and CDH1 expression and is involved in the regulation of phosphorylation of AKT1, RPS6KB1, MTOR, CXCL5, MAPK8, MAP2K1/2, and MAPK3/1 52-54. In addition, MALAT1 may contribute to chemoresistance by driving expression of MDR1 (ABCB1) and MRP1 (ABCC1) through the phosphorylation activation of STAT3 55. MALAT1 may also regulate expression of important genes through a small non-coding RNA-mediated mechanism. For instance, MALAT1 increases zinc finger E-box binding homeobox 1 (ZEB1) expression by sponging miR-200a in A549 cells and promotes the cell proliferation 56. MALAT1 also inhibits miR-200b function in DTX (docetaxel)-resistant lung adenocarcinoma cells. In cytoplasm, MALAT1 weakens the binding of miR-200b to E2F transcription factor 3(E2F3) and ZEB1 mRNAs, thus leading to increase of E2F3 and ZEB1 protein expression and chemoresistance of lung adenocarcinoma cells 57. The regulatory roles of MALAT1 are summarized in Figure 1.

In summary, MALAT1 plays an important role in the development and progression of lung cancer through multiple mechanisms and thus may serve as a potential biomarker and target for treatment of lung cancer. Further study in clinical translation of MALAT1 is warranted.

### 3.2. HOX Transcript Antisense RNA (HOTAIR)

HOTAIR is located on chromosome 12q13.13 in humans and has a length of 2.2 kb containing 6 exons 58. There are four HOX gene clusters (HOXA, HOXB, HOXC, and HOXD) and 39 HOX gene family members in the genome 59. The HOTAIR is a transcript of the antisense strand of HOXC, specifically located between HoxC11 and HoxC12, and may regulate gene expression in HOX loci in a cis- or trans-action manner. Abnormal expression of HOTAIR has been reported in a variety of cancerous tissues, such as lung cancer, pancreatic cancer, breast cancer, colorectal cancer, liver cancer and gastric cancer 60, 61.

In lung cancer, HOTAIR expression is significantly higher in tumor tissues than in the adjacent non-tumor tissues, and the HOTAIR expression was correlated with advanced pathological stage, lymph node metastasis, and poor prognosis, being a negative prognostic factor 62, 63. *In vitro*, HOTAIR regulates apoptosis and cell cycle and is involved in cisplatin resistance of human lung adenocarcinoma cells 64.

**Figure 2:**
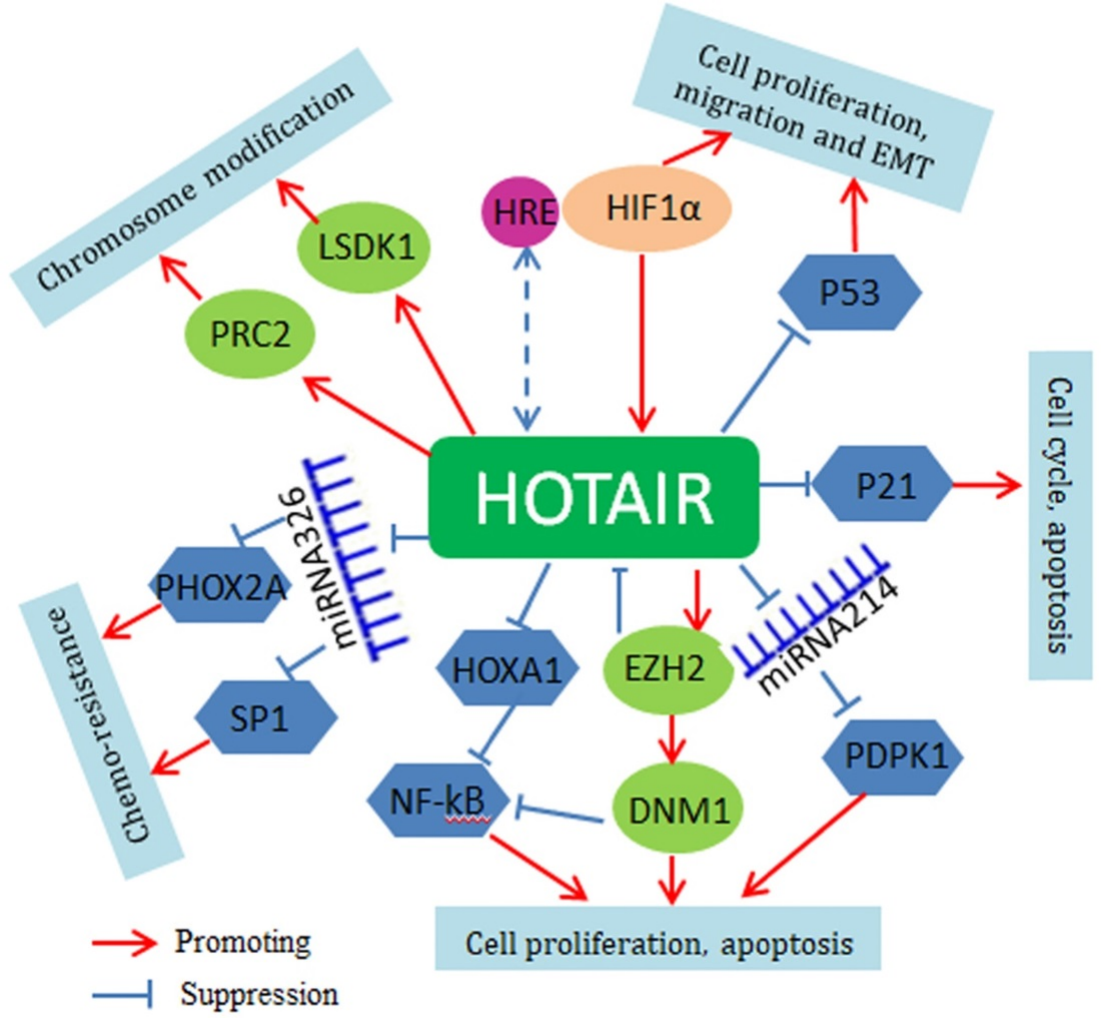
** Regulatory network of HOTAIR in lung cancer.** HOTAIR regulates the growth and proliferation of lung cancer cells by inhibition of p53 and p21. HOTAIR-mediated epigenetic gene expression is dependent on its function as a scaffold for PRC2 and LSD1regulating PTEN, HOXD10, β-catenin and Wif1. Through the methylation of HOXA1, HOTAIR activates the NF-kB signaling pathway. HOTAIR also acts as a ceRNA for regulation of miRNAs, such as miR-329 and miR-214.

Mechanistically, HOTAIR serves as a bridge scaffold for histone modification complexes mediating histone methylation and chromosomal remodeling. Polycomb repressive complex 2 (PRC2) is a histone methyltransferase implementing epigenetic silencing. The 5′ end of HOTAIR binds to PRC2, regulating its occupancy and histone H3 lysine 27 trimethylation at different genes in the genome. The 3' end of HOTAIR can bind to the LSD1/CoREST/REST complex, regulating lysine 4 demethylation 65. Through histone H3 lysine 27 trimethylation of p53, HOTAIR suppresses p53 expression and promotes cell proliferation and invasion 66. HOTAIR can also activate the Wnt/β-catenin signaling pathway to promote tumorigenesis 67. Very recently, it is found that HOTAIR may function through small non-coding RNAs. For instance, HOTAIR interacts with miR-34a-5p to mediate drug sensitivity in lung cancer cells 68. HOTAIR also regulates miR-613 and miR-221 and affects the apoptosis, tumorigenesis and metastasis of NSCLC cells 69, 70. At upstream, HOTAIR is the direct target of HIF-1a and is upregulated under hypoxic conditions; there is a hypoxia-responsive element (HRE) in the HOTAIR promoter region where HIF-1a binds 71. The regulatory mechanisms of HOTAIR are summarized in Figure 2.

In summary, HOTAIR has become as an important novel master regulator of gene expression and lung cancer development and possesses tremendous potentials in the management of this malignancy. Materialization of HOTAIR's clinical potentials in lung cancer, however, requires further investigations.

### 3.3. H19

H19 is a 2.3 kb intergenic and maternally-expressed lncRNA. The H19 gene is located on chromosome 11p15.5, which includes five exons and four introns. H19 is the first imprinted genes identified and evolutionarily conserved in mammals. H19 plays an important role in embryonic development and tumorigenesis, and is associated with multiple cancers, such as lung cancer, bladder cancer, ovarian cancer and pancreatic cancer 72.

H19 expression is significantly higher in lung cancer tissues than in adjacent normal tissues, and interestingly, H19 is elevated in the plasma of lung cancer patients 73. In lung cancer patients, H19 expression is associated with advanced tumor-node-metastasis (TNM) stages, reduced disease-free survival (DFS) and poor prognosis; and in the NSCLC, H19 interacts with microRNA-p21 promoting cancer progression and worse prognosis 73, 74.

In culture cells, H19 promotes cell proliferation, migration, invasion, and epithelial-mesenchymal transition (EMT), but inhibits apoptosis. H19 can upregulate zinc finger E-box binding Homeobox1 and 2 (ZEB1 and ZEB2) through inhibition of miR-200a function and thus promotes cell proliferation, invasion and EMT 75. H19 also induces cell proliferation through promoting the expression of proto-oncogene LIN-28 and inhibiting the homologous gene B (LIN-28B) in lung cancer cells A549 and H1299 76. In addition, H19 promotes the migration and invasion of NSCLC cells through regulation of cellular signaling pathway proteins, such as metastasis associated in colon cancer1 (MACC1), epidermal growth factor receptor (EGFR), β-catenin, and extracellular-signal-regulated kinase1/2 (ERK1/2) 77.

H19 expression is regulated by c-Myc and p53. The oncogene c-Myc activates the transcription of H19 through binding to its promoter 78, 79. Tumor suppressor p53 and H19 are mutually counter-regulated, in which p53 represses the H19 expression while the H19 inhibits p53 and p53-dependent gene expression. The p53-H19 interplay appears to play an important role in tumorigenesis and metastasis. In summary, H19 promotes the progression of lung cancer through multiple mechanisms and may also serve as a serological biomarker. Targeting H19 in lung cancer may represent a novel strategy for the diagnosis and management of this malignancy.

### 3.4. CDKN2B Antisense RNA 1 (CDKN2B-AS1 or ANRIL)

CDKN2B antisense RNA 1 (CDKN2B-AS1), also called ANRIL, is an RNA gene localized on cytogenetic band 9p21.3 with a length of 126.3 kb, consisting of 19 exons 80. The ANRIL transcript is 3.8 kb. ANRIL is involved in multiple cancers, such as lung cancer, breast cancer, and liver cancer 80-82.

ANRIL expression is increased in NSCLC tumor tissues, and its expression levels are significantly correlated with tumor size, lymph node-metastasis and poor prognosis. In culture cells, siRNA-mediated knockdown of ANRIL expression inhibits the cell proliferation and promote apoptosis 81. In NSCLC cells, ANRIL promotes proliferation and inhibits apoptosis through suppressing KLF2 and p21 transcription 81. ANRIL also modulates activity of E2F3 through regulation of miR-449a, leading to cell cycle arrest and senescence of NSCLC cells 83. Recent studies also showed that ANRIL may drive LAD chemo-resistance through regulating the expression of apoptotic related proteins in LAD cells A549, such as cleaved PARP and Bcl-2 84. ANRIL is a downstream effector of c-Myc. The c-Myc directly binds to the E-box in the promoter region of ANRIL and induces ANRIL expression 85. In brief, ANRIL is involved in the development and progression of lung cancer and further study is warranted.

### 3.5. Actin Filament Associated Protein 1 Antisense RNA 1 (AFAP1-AS1)

AFAP1-AS1 was initially identified in esophageal cancer. It is a 6.8 kb antisense lncRNA, and its gene is located on chromosome 4p16.1. The exon 2 of AFAP1-AS1 gene overlapped with exons 14, 15 and 16 of AFAP1 gene, and thus regulates the expression of AFAP1. The AFAP1-AS1 is upregulated in many malignant tumors, including lung cancer, HCC, ovarian cancer, gastric cancer, and colorectal cancer 86.

The expression of AFAP1-AS1 is significantly higher in NSCLC tissues than that in adjacent normal tissues, and AFAP1-AS1 expression is positively correlated with tumor pathological grades, TNM stages and distant metastasis of NSCLC, as well as the clinical outcomes of NSCLC patients 87, 88. AFAP1-AS1 may exert an oncogenic role in the NSCLC cells through epigenetic suppression of p21 expression and serve as a novel prognostic biomarker in human NSCLC 89, 90.

### 3.6. Urothelial Carcinoma Associated 1 (UCA1)

UCA1 is a 2.3 kb lncRNA located in human chromosome 10p13.12. UCA1 is originally cloned from the human bladder cell lines, and is considered to function as a biomarker for the bladder cancer 91. Several studies have suggested that UCA1 expression is increased in multiple types of cancers, include lung cancer, breast cancer, gastric cancer, and colorectal cancer 91.

UCA1 transcript is elevated in NSCLC tissues and promotes disease progression, and the UCA1 expression is negatively correlated with the overall survival in NSCLC 92. Functionally, UCA1 directly regulates miR-193a to increase the expression of HMGB1 that functions as a tumorigenic factor 93, 94. In addition, UCA1 knockdown upregulates the expression of E-cadherin and decreases the expressions of β-catenin, cyclin D1, and MMP-7, indicating that UCA1 promotes EMT and cell invasion partly through β-catenin 95. UCA1 may also induce drug resistance. In tamoxifen resistant breast cells, UCA1 is significantly increased, and inhibition of UCA1 improves tamoxifen sensitivity 96, 97.

## 4. Tumor suppressive lncRNAs in lung cancer

### 4.1. Maternally Expressed Gene 3 (MEG3)

MEG3 is a RNA transcript with 6.9 kb in length and the gene is located on chromosome 14q32.2. MEG3 is the first lncRNA that has been found to have tumor suppressive function. MEG3 is expressed in many normal tissues, but is downregulated in a variety of human tumor tissues 98. MEG3 plays a tumor suppressive role by activating the p53; ectopic expression of MEG3 activates p53 and inhibits tumor growth. The regulation of MEG3 on p53 includes two aspects. On the one hand, MEG3 affects the p53 protein expression via acting as a transcriptional synergistic activating factor. On the other hand, MEG3 affects the half-life of the p53 protein, blocking the degradation of p53 mediated by MDM2 99. MEG3 also inhibits the expression of apoptosis inhibitory protein B-cell lymphoma-2 (BCL2), but promotes the expression of apoptosis promoting factor BCL2-associated X (Bax), thus inducing cell apoptosis. Therefore, MEG3 demonstrates a suppressor function in multiple types of cancers, including hepatocellular carcinoma, glioma, meningioma, neuroblastoma, bladder cancer and hematological malignancies 98.

In NSCLC tissues, MEG3 is downregulated; and the expression of MEG3 is lower in lung cancer cell lines A549 and HCC823. MEG3 promoter methylation is found in most of NSCLC tumor tissues, which mainly contributes to its downregulation 100. In NSCLC tissues, MEG3 expression is negatively correlated with advanced pathological stages and tumor size while high expression of MEG3 in NSCLC tissues is associated with better prognosis, serving as a prognostic factor of NSCLC 92. In NSCLC cells, MEG3 silencing promotes cell proliferation and EMT 99. In addition, MEG3 interacts with miR-21-5p as a molecular sponge and then regulates the sensitivity of NSCLC cells to cisplatin 101. In summary, MEG3 plays an important role in the development, progression and drug sensitivity of lung cancer, and maybe a new prognostic marker and potential therapeutic target for this malignancy.

### 4.2. Growth Arrest Specific 5 (GAS5)

GAS5 gene is located on chromosome 1q25 in humans, and GAS5 lncRNA has a length of 0.65 kb. Evidence to date indicates that GAS5 functions through competitively binding to the glucocorticoid receptor, and thus plays an important role in cell apoptosis 102. GAS5 is associated with the development of a variety of malignancies, including lung cancer, breast cancer, renal cancer, and prostate cancer 103, 104.

In NSCLC, decreased expression of GAS5 is associated with advanced TNM stages and increased tumor size 105. In NSCLC cells, increased expression of GAS5 deregulates E2F1 and drives the expression of p21 and p53, thus inhibiting cell proliferation and promoting apoptosis 106. In addition, GAS5 can deregulate the expression of phospho-EGFR, phospho-MAPK1, phospho-AKT1, and IGF1R; and GAS5 overexpression inversely correlates with the activation of the EGFR pathway 107.

GAS5 can also interact with miRNAs. For instance, miR-21 binds to the putative binding site in GAS5 and regulates its activity; in turn, GAS5 suppresses miR-21 expression as a feedback loop 108. Additionally, GAS5 can sequester hsa-miR-135b-5p and hsa-miR-23a in NSCLC cells 109, 110. GAS5 can also regulate radiosensitivity in NSCLC cells through the PTEN signaling pathway 111. Downregulation of GAS5 promotes resistance to gefitinib in LAD cells 107.

### 4.3. Taurine Up-regulated gene 1 (TUG1)

TUG1 is a 7.1 kb intergenic lncRNA encoded by the gene on chromosome 22q12.2. TUG1 was first discovered as a crucial player in mouse retinal development 112-114. To date, TUG1 is found to functions in a variety of tumors, including lung cancer, HCC, breast cancer, ovarian cancer, bladder cancer, gastric cancer and colorectal cancer 115-118.

TUG1 is dysregulated in multiple cancers and affects cell proliferation and survival 119. TUG1 functions as a tumor suppressor in human glioma through promoting apoptosis and inhibiting cell proliferation 120. TUG1 also acts as a tumor suppressor in NSCLC. A study analyzed 192 NSCLC and adjacent tissues and found that TUG1 expression was downregulated, and low expression of TUG1 was closely related to high TNM stage, tumor size and poor overall survival rate 112.

TUG1 regulates the expression of growth control genes by binding to polycomb repressive complex 2 (PRC2); the TUG1/PRC2 complex could also bind with hoxb7 promoter and epigenetically activate its expression, thereby activating the Akt and mitogen-activated protein kinase (MAPK) pathways in NSCLC 112, 121. TUG1 can also regulate the expression of LIMK2b (a splice variant of LIM-kinase 2) through binding to the enhancer of zeste homolog 2 (EZH2), promoting cell growth and chemoresistance of SCLC 122. TUG1 also competitively binds with transcription factors through the ceRNA function pattern 112-114. In brief, TUG1 is a novel player in lung cancer and may be a potential marker for the management of this malignant disease.

## 5. Conclusive Remarks

LncRNAs have become the key regulatory factors in development and progression of lung cancer, functioning as oncogenes (e.g., MALAT1, HOTAIR, H19 and ANRIL) or tumor suppressors (e.g., MEG3, GAS5, and TUG1). These lncRNAs are up or down regulated in lung cancer tissues and significantly correlated with pathological stages, metastasis and patient survival. Targeted expression or silencing of the lncRNAs in lung cancer cells affect the cell proliferation, migration and invasion, and induce EMT changes and drug resistance. Some lncRNAs, such as H19, are detected and elevated in the circulation blood of lung cancer patients. Therefore, lncRNAs hold a great potential as biomarkers and targets in the management of lung cancer. However, most lncRNAs identified thus far lack lung cancer specificity, and are found to be aberrantly expressed in multiple cancers. This intrinsic limitation may restrict their values in clinical practice. In addition, lncRNAs function in a variety of cell activities and are mechanistically very complex in pathogenic process of lung cancer. Vast efforts are warranted to elucidate the function of lncRNAs in lung cancer and bring them into clinical applications. In addition, only a small portion of lncRNAs have been identified and characterized; most lncRNAs encoded by the genome remain unknown. Efforts are continuously warranted to discover novel lncRNAs and their function in various cancers and subtypes. Nevertheless, lncRNAs are critical, functional molecules in the development and progression of lung cancer and other malignancies, and further study is warranted and may bring a novel paradigm in lung cancer management in near future.

## Figures and Tables

**Table 1:** LncRNAs involved in etiology, diagnosis, treatment and prognosis of lung cancer.

LncRNAs	Involved function	References
MALAT1	Prognosis	1
	Treatment	2, 3
HOTAIR	Etiology	4
	Prognosis	5, 6
	Treatment	7, 8
H19	Etiology	9, 10
	Prognosis	11, 12
	Diagnosis	11
ANRIL	Prognosis	13
	Treatment	13
AFAP1-AS1	Prognosis	14, 15
UCA1	Prognosis	16
	Treatment	17, 18
MEG3	Prognosis	16, 19
	Treatment	20
GAS5	Prognosis	21
	Treatment	22, 23
TUG1	Prognosis	24
	Treatment	25

**Table 2:** Oncogenic and tumor suppressive lncRNAs in lung cancer.

LncRNAs	Chromosome Location	Expression	Key Factors	Function	References
MALAT1	11q13.1	Up	SR, PC2, hnRNP C	Promote cell proliferation, migration, invasion, cell cycle, and EMT; inhibit DNA damage, apoptosis, autophagy	43, 45, 46, 50, 51, 55, 56
HOTAIR	12q13.13	Up	PRC2, LSD1	Promote viability, proliferation, cell cycle, migration, invasion, autophagy, and EMT; suppress apoptosis	62-64, 66, 67, 69, 70
H19	11p15.5	Up	c-Myc, p53, miR-675	Suppress apoptosis; regulate CSCs characteristics; enhance proliferation, differentiation, migration, invasion, and EMT	73-77
ANRIL	9p21.3	Up	PRC2	Enhance cell proliferation, migration, invasion; suppress apoptosis	81, 83, 84
AFAP1-AS	4p16.1	Up	/	Correlate with TNM stages and tumor size	87, 89, 90
UCA1	10p13.12	Up	/	Promotes cell proliferation migration, and EMT	96, 97
MEG3	14q32.2	Down	P53	Inhibit cell viability, proliferation, autophagy, and chemoresistance; induce apoptosis	98-101
GAS5	1q25.1	Down	P53, E2F1, miR-21	Induce apoptosis, drug resistance	105-107, 111
TUG1	22q12.2	Down	PRC2	Suppress cell proliferation, migration; inhibit cell cycle	112, 116, 119-122

## Abbreviations

AFAP1-AS1: actin filament associated protein 1 antisense RNA1
ANRIL: CDKN2B antisense RNA 1
Bax: Bcl2-associated X
Bcl2: B-cell lymphoma-2
CELF1: Elav-like family member 1
CTL: cytotoxic T lymphocyte
DFS: disease-free survival
EGFR: epidermal growth factor receptor
EMT: epithelial-mesenchymal transition
ERK1/2: extracellular-signal-regulated kinase 1/2
GAS5: growth arrest specific 5
HCC: hepatocellular carcinoma
HOTAIR: HOX transcript antisense RNA
HREs: hypoxia-responsive elements
LAD: lung adenocarcinoma
LCC: large cell carcinoma
LncRNAs: long non-coding RNAs
LSCC: lung squamous cell carcinoma
MACC1: metastasis associated in colon cancer 1
MALAT1: metastasis-associated lung adenocarcinoma transcript 1
MAPK: mitogen-activated protein kinase
MDSCs: myeloid-derived suppressor cells
MEG3: maternally expressed gene 3
NSCLCs: non-small cell lung cancer
PBMCs: peripheral blood mononuclear cells
OS: overall survival
PRC2: polycomb repressive complex 2
SCLC: small cell lung cancer
SNP: single nucleotide polymorphism
SOX7: SRY-box transcription factor 7
TKIs: tyrosine kinase inhibitors
TNM: tumor-node-metastasis
TUG1: taurine upregulated gene 1
UCA1: urothelial carcinoma associated 1
ZEB1: zinc finger E-box binding Homeobox 1
ZEB2: zinc finger E-box binding Homeobox 2
